# Action of Mercury

**Published:** 1868-10

**Authors:** 


### Action of Mercury.

At the recent meeting of the British Medical Association in Oxford, Professor Hughes Bennett read an abstract of the results which had been arrived at by the Edinburgh Committee. The committee, after a laborious investigation on the action of mercurials on dogs, arrived at the conclusion, that whether administered in large or small doses, the preparations of mercury exert no cholagogue action upon that animal—in fact, that they always diminish the flow of bile. How far this report can serve to throw light upon the action of mercurials on man, is, however, a matter upon which more than one opinion can be held. In the course of their investigations the committee have found that mercurials, when administered in large doses to dogs, purge them; and, when in smaller and frequently repeated doses, induce the same group of phenomena which are observed in men under the same circumstances, viz.: fetor of the breath, salivation, and ulcerations of the gums. Having accurately ascertained these facts, the committee appear to consider that the fact that mercurials fail to increase the flow of bile in the dog, affords an almost positive proof that these drugs do not exert a cholagogue action in the case of man. The experiments supported also the modern view that the diversion of the bile through a fistulous opening out of the body does not materially interfere with the intestinal functions, but leads to exhaustion of the body altogether. Dr. B. W. Richardson accepted the report as a model of scientific work, but urged still that mercury did exert a beneficial effect, and that experience confirmed its value. Was it possible, he asked, that mercury acted on the pancreatic gland as it did on the salivary glands, and that it caused an increase of pancreatic secretion? Dr. Bennett, in reply, said it was quite possible the pancreatic function was modified under the action of mercury, for, as one of the tables indicated, the pancreas in five cases was reported as very vascular.—*Medical News and Library.*

